# *In Vitro* Antibacterial Activity and Mode of Action of *Piper betle* Extracts against Soft Rot Disease-Causing Bacteria

**DOI:** 10.1155/2023/5806841

**Published:** 2023-09-19

**Authors:** Punyisa Charirak, Rapeepun Prajantasan, Kantapon Premprayoon, Nikom Srikacha, Khakhanang Ratananikom

**Affiliations:** ^1^Department of Plant Production Technology, Faculty of Agricultural Technology, Kalasin University, Kalasin, Thailand; ^2^Department of Science and Mathematics, Faculty of Science and Health Technology, Kalasin University, Kalasin, Thailand; ^3^Department of Agricultural Machinery Engineering, Faculty of Engineering, Rajamangala University of Technology Isan, Khon Kaen Campus, Khon Kaen, Thailand; ^4^Department of Animal Science, Faculty of Natural Resources, Rajamangala University of Technology Isan, Sakon Nakhon Campus, Sakon Nakhon, Thailand

## Abstract

Soft rot disease affects a range of crops in the field and also during transit and storage, resulting in significant yield losses and negative economic impacts. This study evaluated the *in vitro* antibacterial activities and mode of action of *Piper betle* extracts against the soft rot disease-causing bacteria, *Erwinia caratovora* subsp. *caratovora* (*ECC*). Dried leaves of *P. betle* were extracted with water, ethanol, and hexane solvents and evaluated for their antibacterial activity. The results showed the highest antibacterial activity against *ECC* in the ethanol extract, followed by hexane and water extracts with minimum inhibitory concentration (MIC) 1.562, 6.25, and more than 12.50 mg/mL, respectively. The time-kill assay indicated a bactericidal mode of action. *ECC* growth was destroyed within 6 and 8 hours after treatment with the ethanol extract at 4-fold MIC and 2-fold MIC, respectively. The ethanol extract of *P. betle* showed promising activity against *ECC*, with the potential for further development as a novel alternative treatment to control phytobacteria.

## 1. Introduction

Bacterial soft rot is one of the most serious global plant diseases that affects crops both in the field and during transit and storage. This disease devastates a wide variety of crops including potato [1, 2], dragon fruit [3], cabbages [4], cucumbers [4], and tomato [5], with an estimated 15–30% reduction in agricultural production per annum [4].

Bacteria associated with soft rot disease have been extensively studied due to their huge negative economic impact. *Erwinia caratovora* subsp. *caratovora* (*ECC*), a Gram-negative pectolytic bacteria, belongs to the Pectobacteriaceae family and causes softening, wetting, and rotting of internal plant tissues. This pathogen spreads through drain water, sprinkler irrigation, and also manually. It can survive on field weeds and plant debris for a long time, resulting in difficulties in prevention and control [1, 4]. The pathogen also spreads during crop transportation and storage. *ECC* accesses plant tissues through wounds and natural openings such as stomata and, subsequently, produces accumulative amounts of plant cell wall-degrading enzymes such as cellulase, protease, pectate lyase, polygalacturonase, and pectin-methylesterase. These enzymes then degrade plant cell walls, leading to extensive plant tissue maceration and the development of infection. Symptoms have similar appearances on each host, beginning with water-soaked lesions that rapidly enlarge to other areas, often accompanied by an offensive smell [6, 7].

Several strategies are employed to prevent soft rot disease such as plant rotation, improvement of farm management, and selection of healthy crops for culture or storage. However, none of these remedies are entirely successful and are both time- and labor-consuming [2]. Chemicals and antibiotics are also used to control disease distribution in agricultural fields, but this method is expensive and negatively impacts the environment, while also leading to bacterial resistance and health concerns. Chemicals and antibiotics are also unsuitable and not allowed for organic agriculture [8]. Therefore, alternative sustainable methods to control *ECC* are required. Medicinal plants have long been used to manage microbial diseases with promising results. These plants are inexpensive, have reduced side effects, and cause less environmental pollution. Many studies have shown that medicinal plants contain a broad spectrum of constituents to inhibit bacteria, fungi, and yeast which cause human, animal, and plant diseases [9–11].

*Piper betle*, commonly known as betel vine, belongs to the Piperaceae family. In Thailand, it is called Phu and is widely distributed as a popular ethnomedicine in many Asian countries. *P. betle* leaves are used in traditional medicine to cure skin conditions, treat oral and dental problems, headaches, arthritis, and joint discomfort as well as a mouthwash to curb bad breath. Traditional uses of *P. betle* leaves relate to their antibacterial ability [12–14]. Several investigations have revealed additional drug-related properties of *P. betle* including antioxidant, anti-inflammatory, antimalarial, antidiabetic, and gastro- and hepatoprotective activities [12, 14–16]. Although few studies have assessed the effect of *P. betle* extract on the control of phytopathogenic bacteria and fungi.

Thus, this study examined the *in vitro* antibacterial activity of *P. betle* extracts and the mode of action against *ECC*. The knowledge gained can be used to promote a safe alternative treatment for *ECC* management.

## 2. Materials and Methods

### 2.1. Plant Material and Extraction

Leaves of *P. betle* as 500 g samples were randomly collected from three areas in Srisaket Province, Thailand. A single composite sample was prepared by combining 500 g of samples for each representative area and then homogenizing to obtain a uniform single composite sample. The sample was dried in a hot air oven at 60°C for 3 days before milling. Ground samples were extracted with water, ethanol, and hexane at a ratio of 1 : 10 (sample: solvent) on an orbital shaker at 150 rpm for 24 hours [17]. The extracts were filtrated, evaporated at 60°C by a rotary evaporator, and then kept at 4°C for anti-*ECC* activity testing.

### 2.2. Agar-Disc Diffusion Method

The *ECC* strain used in this study was obtained from the Department of Plant Production Technology, Faculty of Agricultural Technology, Kalasin University, Thailand. Dried extracts were dissolved in dimethyl sulfoxide (Loba, India) at a concentration of 50 mg/mL. Dimethyl sulfoxide was used as a cosolvent to dissolve dried extract because of its destring property. It enhances compound solubility as it is miscible with water and organic solvents, and it is not toxic to the test organism [18]. The agar-disc diffusion method was performed for anti-*ECC* activity screening according to the method described by Charirak and Ratananikom (2022) with some modifications [17]. Briefly, sterile blank discs 6 mm in diameter were placed on nutrient agar plates covered with 100 *μ*L of *ECC*. Ten microliters of each extract were allowed to penetrate through the sterile discs into the *ECC*. The plates were then incubated at 30°C for 24 hours. The determination of antibacterial activity was performed as five replicates. The inhibition zone diameter of each extract against *ECC* was measured in millimeters. Dimethyl sulfoxide was used as a negative control and kanamycin (30 *μ*g/disc) (Thermo Scientific, England) was used as a positive control [19].

### 2.3. Minimum Inhibitory Concentration

The minimum inhibitory concentration (MIC) of each extract was evaluated by the resazurin microtiter assay [20] because the resazurin microtiter assay is more sensitive than colorimetric assay such as the MTT assay and XTT assay in measuring cell viability [21]. One hundred microliters of nutrient broth were pipetted into each well of a 96-well plate. A two-fold dilution was performed to prepare the extracts in various concentrations. One hundred microliters of *ECC* culture were added to the mixture of the extracts. The 96-well plates were incubated at 37°C for 24 hours, and then 30 *μ*L of 0.02% resazurin was added. The plates were further incubated at 37°C for 16–18 hours to obtain the MIC, defined as the lowest concentration of extract with no color change of resazurin, indicating the lowest concentration of *P. betle* extract that inhibited microbial growth [20].

### 2.4. Time-Kill Assay

The ethanol extracts were subjected to time-kill curve analysis following Su et al. (2015) with some modifications [22]. An inoculation of 10^6^ CFU/mL of *ECC* culture, harvested from a colony grown overnight, was mixed with ethanol extracts at final concentrations of 2 and 4-fold MIC. One hundred microliters of the mixture were taken at 0, 2, 4, 6, 8, 10, and 12 hours, serially diluted in nutrient broth, and then plated on nutrient agar. After 24 hours of incubation, the number of colonies was counted to determine the total number of visible bacteria. All procedures were performed in triplicate, and a graph of log_10_ (CFU/mL) was plotted against time. Growing cells of *ECC* in nutrient broth without the addition of ethanol extract was used as the control.

### 2.5. Statistical Analysis

The data was expressed as the mean ± standard deviation (S.D.). Statistix version 8 was used for statistical analysis. Statistical analysis was carried out using one-way analysis of variance (ANOVA), with differences between means calculated using the least significant difference (LSD). In all cases, *p* < 0.05 was considered significant.

## 3. Results

### 3.1. Antibacterial Screening

**Figure 1:**
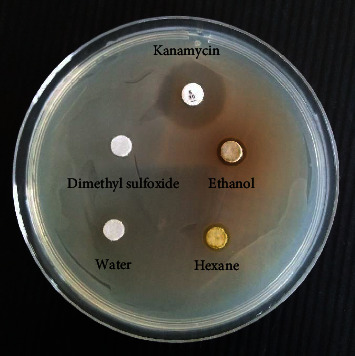
Anti-*ECC* screening of *P. betle* extracts.

Figure 1 and Table 1 show the results of anti-*ECC* screening by agar-disc diffusion. Anti-*ECC* activities showed different degrees of efficiency in all extracts. Kanamycin as the positive control gave the highest anti-*ECC* activity with an inhibition zone of 17.72 ± 0.15 mm, while dimethyl sulfoxide as the negative control showed no inhibitory effect against *ECC*. The ethanol extract gave the second most efficient anti-*ECC* activity (8.48 ± 0.69 mm), followed by the hexane and water extracts at 7.89 ± 0.74 and 7.07 ± 0.74 mm, respectively.

### 3.2. MIC Determination

Table 2 demonstrates the MICs of *P. betle* extracts against *ECC*. The MIC results showed different values and agreed with those from antibacterial screening by agar-disc diffusion. The ethanol extract exhibited the highest activity against *ECC* with the lowest MIC at 1.562 mg/mL, followed by the hexane extract with a MIC 6.250 mg/mL. Conversely, the highest concentration of water extract showed no inhibitory effect toward the *ECC* culture.

### 3.3. Mode of Action of *P. betle* Extracts against ECC

**Figure 2:**
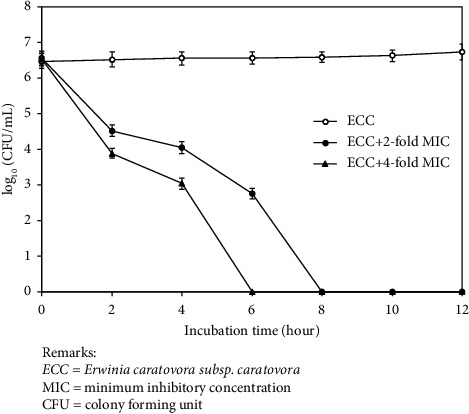
Time-kill curve of ethanol extracts of *P. betle* against *ECC.* Remarks: *ECC* = *Erwinia caratovora subsp. caratovora*, MIC = minimum inhibitory concentration, and CFU = colony forming unit.

Ethanol extracts of *P. betle* at concentrations of 2-fold MIC and 4-fold MIC were used to study its mode of action. The growth curve of *ECC* increased continuously over time, while a remarkable decline was found with incubation of *ECC* with the ethanol extracts. *ECC* viability was completely destroyed within 6 and 8 hours after incubation with the ethanol extracts at concentrations of 4-fold MIC and 2-fold MIC, respectively (Figure 2).

## 4. Discussion

Herbal extracts are a great alternative to harmful chemicals and a safe, eco-friendly way to prevent plant diseases [14, 16]. The identification of an efficient plant extract against *ECC* was conducted. Relatively few botanical extracts have been reported to control soft rot pathogens, while numerous studies have shown the antibacterial action of herbal extracts against many other bacterial pathogens [23, 24].

Results demonstrated that the ethanol extract of *P. betle* significantly inhibited the growth of soft rot bacteria *in vitro*. The MIC of the ethanol extract was 1.562 mg/mL, and it behaved in a bactericidal manner. Interestingly, the time-kill kinetics of the ethanol extract were dose-dependent. *ECC* were completely destroyed within 6 and 8 hours when utilizing ethanol extracts at concentrations of 4-fold MIC and 2-fold MIC, respectively. This result concurred with previous studies indicating that plant crude extracts had bactericidal or bacteriostatic properties. The mode of action of the antibacterial agents was identified using the MBC/MIC ratio or time-kill kinetic [17, 25, 26]. If the ratio of MBC/MIC is ≤4, it is defined as a bactericidal agent, with a bacteriostatic mode of action considered when the ratio is ≥4. In this study, the bactericidal effect of the ethanol extract of *P. betle* was proved by the time-kill assay as a bactericidal agent. This finding was corroborated by earlier studies revealing that essential oil from *P. betle* showed only a bactericidal effect while *P. betle* extracts were both bacteriostatic and bactericidal [14, 27–29]. Differences in mode of action among *P. betle* extracts were attributed to several factors including type of solvent, extraction method, type of indicator strain, dose of the extracts, and phytochemical constituents in the extracts [15].

Significant anti-*ECC* activity of *P. betle* occurred as a result of the synergistic effect of the antibacterial components found in the ethanol extract. One benefit of employing crude plant extracts to suppress pathogenic bacteria is that numerous active ingredients provide a variety of antibacterial actions. A combination of active ingredients is difficult for bacteria to resist; however, synthetic antibacterial agents typically cause bacteria to evolve resistant mechanisms [17]. Our results indicated that the active constituents against *ECC* growth found in *P. betle* showed moderate hydrophilic properties. The most suitable extraction solvent for obtaining the highest anti-*ECC* property was ethanol, with a polarity less than that of water but higher than that of hexane. This result indicated that the extraction solvent also plays an important role in the isolation of active components from plants and was supported by earlier studies that determined organic solvents to be typically more effective at extracting antimicrobial compounds than aqueous-based techniques [14, 17]. Ethanol extracts from *P. betle* also demonstrated excellent antibacterial properties over Gram-positive and Gram-negative bacteria, including those classified as multidrug resistant such as metallo-*β*-lactamase-producing *P. aeruginosa* and *A. baumannii*, MRSA, and VRE [14]. Some phytogenic bacteria and fungi are also inhibited by the extract of *P betle* such as *Xanthomonas axonopodis p.v. malvacearum* [24], *X. axonopodis pv. vericatoria* [24], *Xanthomonas oryzae* [24], *Xanthomonas campestris p.v. campestris* [24], *Fusarium oxysporum* [30], *Plasmopara viticola* [23], and *Candida albicans* [31]. The ethanol extract of the *P. betle* showed a broad spectrum toward pathogenic microbes. The exact chemical constituents in the ethanol extract were not evaluated in this study, although significant chemical components have previously been identified in *P. betle* extracts. Aoki et al. (2019) [23] examined the inhibitory effect of methanol extract from *P. betle* leaves on grape downy mildew. Their results showed that the methanol extract suppressed grape downy mildew pathogens, and they identified four components in the methanol extract including 4-allylpyrocatechol, eugenol, *α*-pinene, and *β*-pinene. Singtongratana et al. (2013) [32] recorded major constituents in *P. betle* extract as hydroxychavicol or allypyrocatechol. These chemical components have been reported for antimicrobial activity against several strains, causing plasma membrane damage, coagulation of nucleoid, plasma membrane permeability alteration, and proton pumping inhibition [14, 15, 31, 33, 34]. Therefore, these components may be found in the ethanol extract due to the relatively similar polarity of the extraction solvent used in this study, leading to *ECC* death.

*P. betle* is ubiquitously found in Thailand and the leaves are easily accessible and cost-free. The decomposition of *P. betle* leaves also contributes to increased soil fertility. Therefore, employing *P. betle* leaf extract has no negative phytotoxin impacts on plants. The success in eliminating *ECC* provides fresh information regarding the most effective way to use *P. betle* extract to manage soft rot disease.

## 5. Conclusion

*P. betle* showed the potential for antimicrobial application *in vitro*. Its ethanol extract demonstrated a bactericidal mode of action to completely inhibit the *ECC*, soft rot disease-causing bacteria. It could be said that this extract can be used as an alternative to chemical bactericides in organic agriculture.

## Figures and Tables

**Table 1:** Inhibition zone diameters of *P. betle* extracts against *ECC*.

Extraction solvent	Inhibition zone diameter (mm)
Water	7.07 ± 0.74^d^
Ethanol	8.48 ± 0.69^b^
Hexane	7.89 ± 0.74^c^
Dimethyl sulfoxide	6.00 ± 0.00^e^
Kanamycin	17.72 ± 0.15^a^

Remarks: different letters in the column indicate significant differences (*p* < 0.01).

**Table 2:** MICs of *P. betle extracts* against *ECC*.

Extraction solvent	MIC (mg/mL)
Water	>12.500
Ethanol	1.562
Hexane	6.250

Remarks: MIC = minimum inhibitory concentration.

## Data Availability

The data used to support the findings of this study are available from the corresponding author upon reasonable request.
